# Phospholamban Ablation Using CRISPR/Cas9 System Improves Mortality in a Murine Heart Failure Model

**DOI:** 10.1371/journal.pone.0168486

**Published:** 2016-12-16

**Authors:** Manami Kaneko, Kentarou Hashikami, Satoshi Yamamoto, Hirokazu Matsumoto, Tomoyuki Nishimoto

**Affiliations:** 1 Cardiovascular and Metabolic Drug Discovery Unit, Pharmaceutical Research Division, Takeda Pharmaceutical Company Limited, Fujisawa, Kanagawa, Japan; 2 Integrated Technology Research Laboratories, Pharmaceutical Research Division, Takeda Pharmaceutical Company Limited, Fujisawa, Kanagawa, Japan; Rutgers New Jersey Medical School, UNITED STATES

## Abstract

Sarcoplasmic reticulum Ca^2+^-ATPase 2a (SERCA2a) and its inhibitory protein called phospholamban (PLN) are pivotal for Ca^2+^ handling in cardiomyocyte and are known that their expression level and activity were changed in the heart failure patients. To examine whether PLN inhibition can improve survival rate as well as cardiac function in heart failure, we performed PLN ablation in calsequestrin overexpressing (CSQ-Tg) mice, a severe heart failure model, using clustered regularly interspaced short palindromic repeat (CRISPR)/CRISPR-associated (Cas) system. According this method, generation rate of PLN wild type mice (PLN copy >0.95) and PLN homozygous knockout (KO) mice (PLN copy <0.05) were 39.1% and 10.5%, respectively. While CSQ overexpression causes severe heart failure symptoms and premature death, a significant ameliorating effect on survival rate was observed in PLN homozygous KO/CSQ-Tg mice compared to PLN wild type/CSQ-Tg mice (median survival days are 55 and 50 days, respectively). Measurement of cardiac function with cardiac catheterization at the age of 5 weeks revealed that PLN ablation improved cardiac function in CSQ-Tg mice without affecting heart rate and blood pressure. Furthermore, increases in atrial and lung weight, an index of congestion, were significantly inhibited by PLN ablation. These results suggest that PLN deletion would be a promising approach to improve both mortality and cardiac function in the heart failure.

## Introduction

Heart failure (HF) is the complicated clinical syndrome characterized by progressive cardiac remodeling and dysfunction. Despite the advances in device therapy [1, 2] and pharmacological therapy such as angiotensin II receptor blocker [3], angiotensin converting enzyme inhibitors [4], and β-blockers [5], HF remains a major cause of morbidity in the world [6, 7]. Additionally, prevalence of HF is growing due to raise of aging population in the developed countries, therefore more novel and effective treatments for HF is required. Research about the mechanism of HF over the year revealed that one of the most consistent cellular features in HF patients is an impaired Ca^2+^ homeostasis with alterations in the amplitude and kinetics of Ca^2+^ transients [8–10]. Ca^2+^ has critical roles as a second messenger in several signaling pathways in the heart, and this abnormal Ca^2+^ handling in HF involves contractile dysfunction, remodeling, abnormal electrical activity, reduction of ATP production, and apoptosis [11–13]. Sarco/endoplasmic reticulum calcium ATPase 2a, (SERCA2a) expressed in sarcoplasmic reticulum (SR) and its inhibitory protein called phospholamban (PLN) are major proteins regulating Ca^2+^ handling. Reductions of SERCA2a protein expression and activity, and an enhancement of inhibitory effect of PLN are associated with impaired SR Ca^2+^ uptake in cardiomyocytes of HF, subsequently decrease Ca^2+^ release from SR to cytosol via ryanodine receptor, which induces the reduction of both systolic and diastolic function and, eventually cardiac death [14–16]. Therefore, SERCA2a and PLN are expected as a target for novel therapy of HF for a few decades, indeed, it was reported that the normalization of SERCA2a function and the PLN inhibition increased contractility with correction of Ca^2+^ homeostasis in a large number of studies in isolated cardiomyocyte and animal models of HF [17–19]. However, positive inotropic agents, such as β-adrenoceptor agonists and PDE III inhibitors, which increase cardiac contractility with increasing cAMP levels and stimulating Ca^2+^ cycling, caused worsening mortality in HF patients as a result of increased energetic consumption and abnormal electrical activity [20, 21]. Although similar concerns are raised regarding SERCA2a activation or PLN inhibition therapy which causes enhancement of Ca^2+^ cycling and contractility, there are few reports about survival in preclinical study. The purpose of this study was to examine the effects of PLN ablation on mortality in the calsequestrin (CSQ)-Tg mouse, a severe HF model showing premature death with abnormal Ca^2+^ handling, and various features similar to HF patients such as hypertrophy, fibrosis, and pump failure [22, 23]. Although the genetic background of BDF1, a cross between DBA/2 mice and C57BL/6 mice, is critical for onset of early cardiac death in CSQ-Tg mice [24, 25], it is difficult to breed BDF1 mice because they died within ten weeks after birth. Thus, offspring from male CSQ-Tg mice with a DBA/2 background and female DBA/2 mice, which survive for a half year, are used for breeding. It takes very long time for conventional gene targeting methods using ES cells because establishments of each ES cell line as well as several times of crossbreeding are required. Therefore, in this study, clustered regularly interspaced short palindromic repeat (CRISPR)/CRISPR-associated (Cas) system known as efficient and simplified genome editing technology [26, 27] was applied for generation of PLN KO/CSQ-Tg mice with microinjection technique using interspecies mouse embryos.

## Materials and Methods

### Preparation of microinjection components

**Fig 1:**
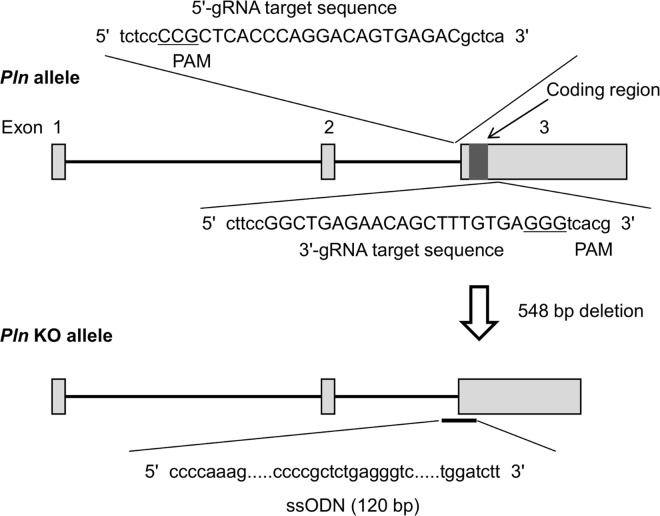
sgRNAs and ssODN targeting *Pln* coding region. Targeting sequences of each sgRNA are capitalized and NGG PAM (protospacer-adjacent motif) sequences are underlined. Exons are indicated by closed boxes. The sgRNA targeting sites were designed to sandwich *Pln* coding region (filled with black). The ssODN is containing homologies of 60 bases on both sides flanking each of the sgRNA targeting sequences.

T7 promoter sequence was added to Cas9 coding sequence by PCR for the preparation of Cas9 mRNA. The amplified PCR fragment was ligated into pMD20-T vector (Takara Bio, Shiga, Japan). The Cas9 plasmid linearized by XbaI digestion was used as a template for *in vitro* transcription using mMESSAGE mMACHINE T7 Ultra Kit (Life technologies, California, USA). This transcription procedure was performed according to the manufacturer’s protocol. The obtained Cas9 mRNA was purified by RNeasy mini kit (QIAGEN, Hilden, Germany). Two sgRNA sequences were designed to sandwich the coding region of the *Pln* gene (Fig 1). T7 promoter was added to each of the sgRNA sequences by PCR. The amplified PCR fragments were used as templates for *in vitro* transcription using MEGAshortscript T7 Kit (Life technologies) according to the manufacturer’s protocol. The sgRNAs were purified by RNeasy mini kit. The single-stranded oligodeoxynucleotide (ssODN) purchased as Standard Oligo (Eurofins Genomics, Tokyo, Japan) (Fig 1).

### Animals

All animal experiments were approved by the Institutional Animal Care and Use Committee (IACUC) of Shonan Research Center, Takeda Pharmaceutical Company Limited. The line of transgenic mice overexpressing canine calsequestrin (CSQ) in the heart was originally established in the Indiana University School of Medicine [22]. The transgenic line was developed on a DBA/2 background. Body weight was measured once a week and survival was monitored once a day. After 7 weeks of age, rectal temperature was checked every day, and mouse which showed less than 29°C was euthanized by carbon dioxide (less than 30%). The mouse with clinical symptoms such as extremely reduced physical activity and body weight loss was euthanized by carbon dioxide. We used 52 mice in the survival study. Twenty nine of mice were euthanized and 23 of mice were died. Necropsy examinations revealed that remarkable hypertrophy was exhibited in almost every carcass. In contrast, there were no obvious changes of other organs such as liver, spleen, stomach, genitals, and brain, while we did not perform pathological analysis in detail. Thus, we consider that the primal cause of death is a heart failure in CSQ-Tg mice. This outcome of mortality in this study was anticipated such that it was reviewed and approved by the IACUC.

We determined the sample size for survival study according to previous papers [28, 29]. The sample sizes for survival study were n = 8 to 28 and n = 12 to 16 per group, respectively, thus, we determined each group includes more than 10 mice for this study. In 4 injections, we were able to obtain 14 male PLN homozygous KO/CSQ-Tg mice (and 38 male PLN WT/CSQ-Tg mice), and conducted the survival study using all of them. In a cardiac function study, general sample sizes including the papers above was n = 5 to 10 per group. Therefore, we used all male PLN homozygous KO/CSQ-Tg mice, n = 6, developed by the 5th injection.

### Microinjection into mouse embryos

For microinjection, zygotes were obtained by *in vitro* fertilization (IVF) of sperm isolated from male CSQ-Tg mice with a DBA/2 background and eggs from female C57BL/6 mice (CLEA Japan, Tokyo, Japan). Cas9 mRNA, two sgRNAs, and ssODN were diluted and mixed in 0.1×TE buffer (10 mM Tris-HCl, 0.1 mM EDTA (pH 8.0)) to a working concentration of 100 ng/μL, 50 ng/μL, and 50 ng/μL, respectively. Then the mixture was injected into the pronuclei of zygotes using a micromanipulator and a microinjector. The manipulated embryos were transferred into the oviducts of pseudopregnant foster mothers (ICR, CLEA Japan).

### Genotyping

Genomic DNA in the ear was extracted from each offspring using a DNeasy Kit or a Puregene Kit (QIAGEN) according to the manufacturer’s protocol. For the screening of PLN knockout mice, the copy number of *Pln* gene was analyzed by quantitative PCR (qPCR) using the genomic DNA as a template. The qPCR was performed using TaqMan Fast Universal PCR Master Mix (Life technologies) and TaqMan MGB probe kit designed on the coding region. All samples were analyzed by 7900HT Fast Real Time PCR system (Life technologies) and normalized against nerve growth factor (*Ngf*). To confirm the deletion of *Pln* coding region, PCR was performed using primers flanking the target region of *Pln* gene (5'-GTC CTT ACT GTG CCT TCT GAG TTT G-3', 5'-GAC TGG AGC TAT AAA GTG ACC TTG C-3'). The following cycle conditions were used: 98°C for 10 s, 60°C for 15 s and 68°C for 10 s/kb for 32 cycles. The PCR products were analyzed by electrophoresis using LabChip GX system (Caliper LifeSciences, Massachusetts, USA). The PCR product sizes were 1213 bp for wild type allele and 665 bp for knockout allele. To identify CSQ-Tg mice, PCR of genomic DNA was carried out using primers corresponding to myosin heavy chain gene sequence and the 5'-end of the canine *Csq* cDNA sequence (5'-CTC TGA CAG AGA AGC AGG CAC TTT ACA TGG-3', 5'-GAT GAA CAG GTG TGT TCT CTT CAT-3'), and the PCR product size was 407 bp. The PCR cycle condition and the electrophoresis of PCR products were performed as described above.

### Hemodynamic parameter measurement

Mice were anesthetized with 1.5–2.5% isoflurane. A catheter with a pressure sensor diameter of 1.4F (SPR-671: Millar Instruments, Texas, USA) was inserted into the left ventricle through the right carotid artery and connected to a polygraph system (NEC San-ei Instruments Ltd. and Nihon Kohden Corporation, Tokyo, Japan). The measurement parameters were mean blood pressure (MBP), heart rate (HR), left ventricular pressure, left ventricular end diastolic pressure (LVEDP), the rates of intraventricular pressure rise (dP/dt_max_) and decline (dP/dt_min_), and tau calculated using Weiss method by fitting a decaying exponential function to part of the left ventricular relaxation curve. The data were incorporated into PowerLab and analyzed by LabChart v.8 and blood pressure module (AD Instruments, Castle Hill, Australia). Immediately after the measurement, the hearts were rapidly excised, and individual chambers were separated, and weighed. These manipulations were conducted under blind condition. After weight measurement, the heart samples were rapidly frozen by liquid nitrogen and preserved at -80°C until western blot analysis and mRNA measurement. One sample in PLN wild type/CSQ non-Tg mice was lost before western blot analysis and mRNA measurement.

### Western blot analysis

Frozen hearts were homogenized in RIPA buffer (Thermo Fisher Scientific, Massachusetts, USA) containing plus proteinase inhibitors and phosphatase inhibitors (Roche, Basel, Switzerland) and centrifuged at 20,000 x g for 10 min at 4°C. After a determination of protein concentrations, SDS-PAGE was performed using the supernatant with CriterionTM Precast Gels (10–20% Tris-Tricine/Peptide, BIO-RAD, California, USA) for PLN and CriterionTM TGXTM Precast Gels (4–20%, BIO-RAD) for SERCA2a and actin. They were transferred to PVDF membranes in the Trans-Blot Turbo (BIO-RAD) and blocked in PVDF Blocking Reagent (TOYOBO, Osaka, Japan). For immunoreaction, blots were incubated with 1: 1000 diluted monoclonal antibody to PLN (MilliporeSigma, Darmstadt, Germany), 1: 1000 diluted monoclonal antibody to SERCA2a (abcam, Cambridge, UK), or 1: 1000 diluted monoclonal antibody to actin (MilliporeSigma) at 4°C overnight. The antigens were detected by the luminescence method (Amersham ECL Prime kit, GE Healthcare, Little Chalfont, UK) with 1: 25000 diluted anti-mouse IgG (GE Healthcare) or 1: 25000 diluted anti-rabbit IgG (GE Healthcare) in the ChemiDoc (BIO-RAD). The intensity of bands was calculated by Image Lab 4.0 (BIO-RAD).

### mRNA expression analysis by real-time PCR

Total RNA was extracted using RNeasy mini kit (QIAGEN), and converted into cDNA with High Capacity cDNA Reverse Transcription Kit (Life Technologies). Gene expression was analyzed by 7900HT Fast Real-Time PCR System (Life Technologies) using TaqMan® Universal Master Mix II (Life Technologies) and primer-probe sets of TaqMan Gene Expression Assays (Life Technologies): Myosin heavy chain beta isoform (β-MHC) (Mm01319006_g1), atrial natriuretic factor (ANF) (Mm01255747_g1), brain (ventricular) natriuretic factor (BNF) (Mm01255770_g1). Beta-actin was used as an endogenous control gene. Relative mRNA expression was calculated by ΔΔCT method.

### Statistical Analysis

Data are expressed as means ± S.D. Survival data were analyzed by using a Kaplan–Meier survival analysis with Gehan-Breslow-Wilcoxon statistics. Analysis was performed with the Student’s *t*-test for other experiments.

## Results

### Generation of PLN KO/CSQ-Tg mice

**Fig 2:**
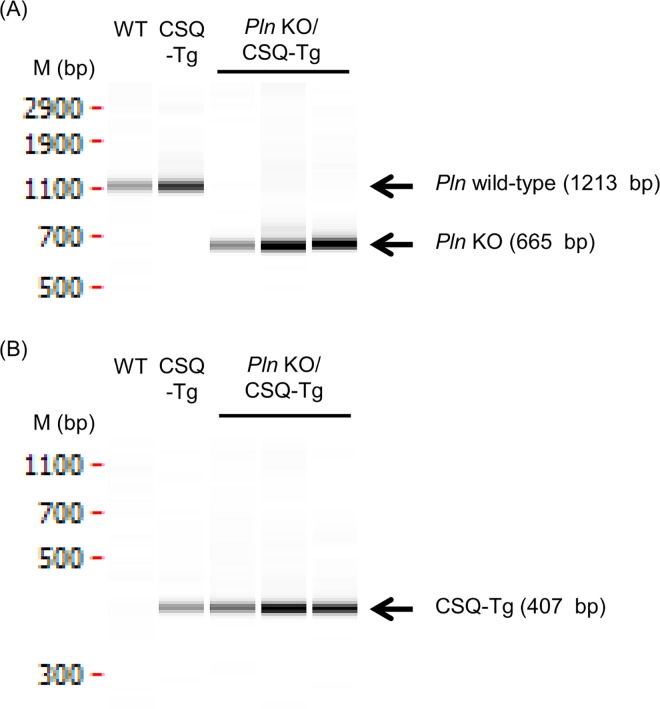
PCR analysis of mice obtained from 1st lot microinjection. (A) Genotyping of PLN KO mice by PCR. PLN wild type (WT) mice showed 1213 bp fragments and PLN KO mice showed 665 bp fragments. (B) Genotyping of CSQ-Tg mice by PCR. Mice which had canine *Csq* allele shows 407 bp fragments and Non-Tg mice does not show any fragments. M, DNA marker.

We used five lots of CSQ-Tg mice given PLN knockout manipulation in our study. CRISPR/Cas mixture was injected into the pronucleus of 4051 zygotes in total and 668 pups were obtained in the five experiments (Table 1). We carried out genotyping them by qPCR to determine the copy number of *Pln* gene. We found that 70 were homozygous knockout (KO) mice lacking coding region of *Pln* gene (*Pln* copy < 0.05) and 261 were wild type (WT) mice (*Pln* copy > 0.95) (Table 1). Generation rate of PLN homozygous KO mice in third, fourth, and fifth lot were higher than those in first and second one. This explanation is that more CRISPR/Cas mixture was injected into the pronucleus of zygotes in these lots. PCR products from these homozygous KO mice showed that they deleted the coding region of *Pln* gene without detectable WT amplicons (Fig 2A). Next, we identified CSQ-Tg mice by PCR of genomic DNA from obtained pups (Fig 2B). 328 out of 668 pups were determined to be CSQ-Tg mice. We finally obtained 40 PLN homozygous KO/CSQ-Tg mice (12.2%, male: 20, female: 20) and 117 PLN WT/CSQ-Tg mice (35.7%, male: 62, female: 55) (Table 2).

**Table 1:** Number of transferred embryos, pups, PLN homozygous KO mice, and PLN wild type (WT) mice in five lots.

Lot	Injected zygotes	Transferred embryos (%)^a^	Pups (%)^b^	PLN homozygous KO (%)^c^	PLN WT (%)^d^
1^st^ lot	660	607 (92.0)	119 (19.6)	8 (6.7)	52 (43.7)
2^nd^ lot	701	649 (92.6)	173 (26.7)	8 (4.6)	55 (31.8)
3^rd^ lot	644	578 (89.8)	87 (15.1)	13 (14.9)	38 (43.7)
4^th^ lot	695	623 (89.6)	109 (17.5)	18 (16.5)	34 (31.2)
5^th^ lot	1351	1228 (90.9)	180 (14.7)	23 (12.8)	82 (45.6)

Values in parenthesis shows generation rate.

^a^Transferred embryos/Injected zygotes

^b^Pups/Transferred embryos

^c^PLN homozygous KO/Pups

^d^PLN WT/Pups.

**Table 2:** Number of CSQ-Tg and Non-Tg mice in five lots.

Lot	Total	CSQ-Tg		Non-Tg	
		PLN homozygos KO	PLN WT	PLN homozygos KO	PLN WT
1^st^ lot	60	4 (3, 1)	18 (11, 7)	4 (2, 2)	34 (22, 12)
2^nd^ lot	63	3 (2, 1)	22 (9, 13)	5 (3, 2)	33 (18, 15)
3^rd^ lot	51	7 (3, 4)	18 (10, 8)	6 (3, 3)	20 (12, 8)
4^th^ lot	52	11 (6, 5)	18 (8, 10)	7 (1, 6)	16 (6, 10)
5^th^ lot	105	15 (6, 9)	41 (24, 17)	8 (2, 6)	41 (20, 21)

Values in parenthesis shows number of male and female mice.

### Mortality

**Fig 3:**
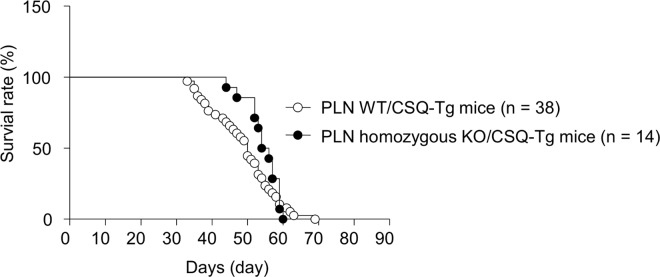
Survival curve in PLN wild type (WT)/CSQ-Tg mice and PLN homozygous KO/CSQ-Tg mice.

Male mice in lot 1–4 were used for survival study. As shown in Fig 3, CSQ-Tg mice started to die from 30 days after birth, finally all of them died within around 60 days as previously reported [22]. While PLN homozygous KO/CSQ-Tg mice also died within 60 days after birth, onset of death was extended to over 40 days after birth. Median survival days of CSQ-Tg mice without and with PLN deletion are 50 and 55 days, respectively. Gehan-Breslow-Wilcoxon statistics showed significant ameliorating effect on survival rate in PLN homozygous KO/CSQ-Tg mice compared to PLN WT/CSQ-Tg mice (p = 0.04). Both PLN WT/CSQ non-Tg mice and PLN homozygous KO/CSQ non-Tg mice were not included in the survival study because they did not show premature death unlike CSQ-Tg background in our preliminary study.

### Hemodynamic parameters

**Fig 4:**
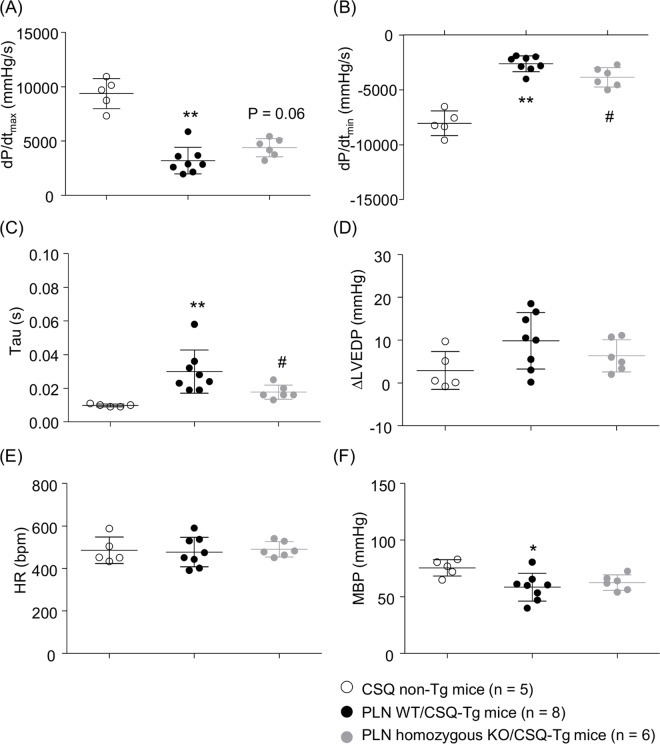
Hemodynamic parameters. Cardiac catheterization was performed in CSQ non-Tg mice, PLN wild type (WT)/CSQ-Tg mice, and PLN homozygous KO/CSQ-Tg mice. (A) dP/dt_max_, (B) dP/dt_min_, (C) Tau, (D) LVEDP, (E) MBP, (F) HR. Values represent the mean ± SD, *P < 0.05, **P < 0.01 vs. CSQ non-Tg mice, ^#^P < 0.05 vs. PLN WT/CSQ-Tg mice by Student's *t*-test.

To determine whether PLN ablation affected hemodynamic parameters, cardiac catheterization was performed in 5-week-old male mice in the lot 5 (Table 2). Compared with PLN WT/CSQ non-Tg mice (showed simply as CSQ non-Tg mice in our study, *Pln* copies; 1.17 ± 0.04, n = 5), PLN WT/CSQ-Tg mice (*Pln* copies; 1.20 ± 0.04, n = 8) had severe cardiac dysfunction, as shown by the marked reductions in both dP/dt_max_ and dP/dt_min_, the prolongation of tau, the tendency of elevation of LVEDP, and the lowering blood pressure (Fig 4). PLN homozygous knockout (*Pln* copies; 0.001 ± 0.001, n = 6) significantly improved diastolic function (dP/dt_min_ and tau), had tendency to improve systolic function (dP/dt_max_) without affecting HR and MBP. Even in mosaic group (*Pln* copies; 0.50 ± 0.14, n = 7), it was shown the trend toward the improvement of diastolic function and systolic function (data not shown). We obtained only two PLN homozygous KO/CSQ non-Tg mice in the lot 5 and failed to measure cardiac function of one mouse. Each value of cardiac function parameters in PLN homozygous KO/CSQ non-Tg mice (n = 1) were dP/dt_max_: 12379 mmHg/s, dP/dt_min_: -9754 mmHg/s, tau: 0.007s, ΔLVEDP: 2.0 mmHg, HR: 411 bpm, and MBP: 70 mmHg, respectively.

### Western blot analysis

**Fig 5:**
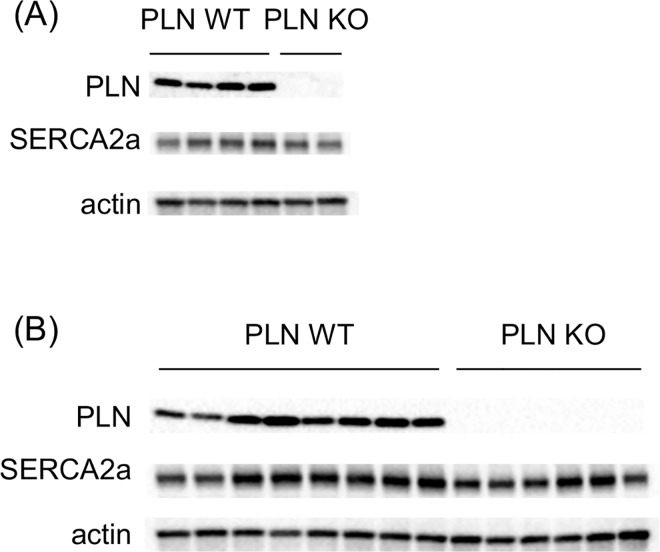
Immunoblots of PLN and SERCA2a, and actin in hearts from mice used for hemodynamic study. (A) CSQ non-Tg mice, (B) CSQ-Tg mice.

Protein levels of PLN and SERCA2a were measured in hearts isolated from mice used for the hemodynamic study. PLN proteins were completely defected by the PLN ablation using CRISPR/Cas system in both CSQ non-Tg and CSQ-Tg mice, as shown in Fig 5. On the other hand, protein levels of SERCA2a were not changed by the PLN ablation (SERCA/actin ratio, PLN WT/CSQ non-Tg mice: 2.00 ± 0.43 (n = 4) vs. PLN KO/ CSQ non-Tg mice: 1.74 (n = 2), PLN WT/CSQ-Tg mice: 1.90 ± 0.30 (n = 8) vs. PLN KO/CSQ-Tg mice: 1.59 ± 0.43 (n = 6)).

### Cardiac and pulmonary weight

**Fig 6:**
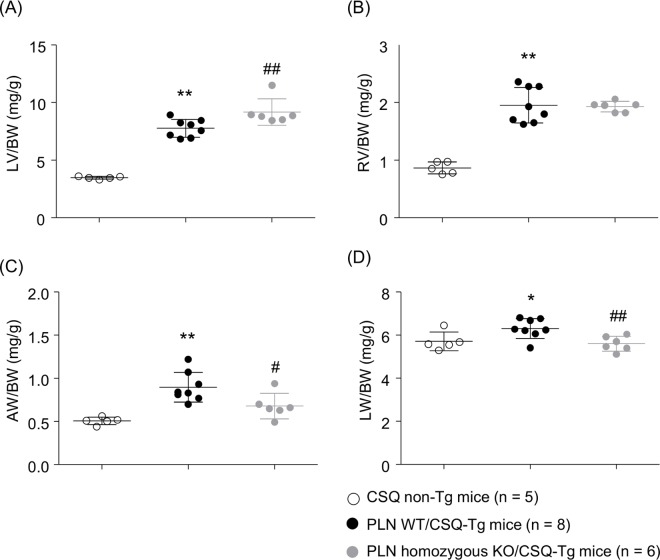
Heart and lung weight. Heart and lung weight were measured in CSQ non-Tg mice, PLN wild type (WT)/CSQ-Tg mice, and PLN homozygous KO/CSQ-Tg mice. (A) LV/BW (left ventricular weight/body weight), (B) RV/BW (right ventricular weight/body weight), (C) AW/BW (atria weight/body weight), (D) LW/BW (lung weight/body weight). Values represent the mean ± SD, *P < 0.05, **P < 0.01 vs. CSQ non-Tg mice, ^#^P < 0.05, ^##^P < 0.01 vs. PLN WT/CSQ-Tg mice by Student's *t*-test.

Ventricular, atrial, and pulmonary weight in the mice used in the hemodynamic study were evaluated to assess whether improvement of cardiac function by PLN ablation was accompanied with reduction of hypertrophy. All of tissue weight/body weight ratios were increased in PLN WT/CSQ-Tg mice compared with CSQ non-Tg mice (Fig 6). PLN ablation significantly suppressed the atrial and pulmonary weight. Interestingly, left ventricular weight was increased rather than decreased in PLN homozygous KO/CSQ-Tg mice. All of tissue weight/body weight ratios in two PLN homozygous KO/CSQ non-Tg mice (n = 2) showed similar trend with PLN homozygous KO/CSQ-Tg mice, which ventricular weight was larger than those in PLN WT mice (LV/BW: 3.95 mg/g, RV/BW: 1.06 mg/g, AW/BW: 0.42 mg/g, LW/BW: 6.15 mg/g, respectively).

### mRNA expression analysis

**Fig 7:**
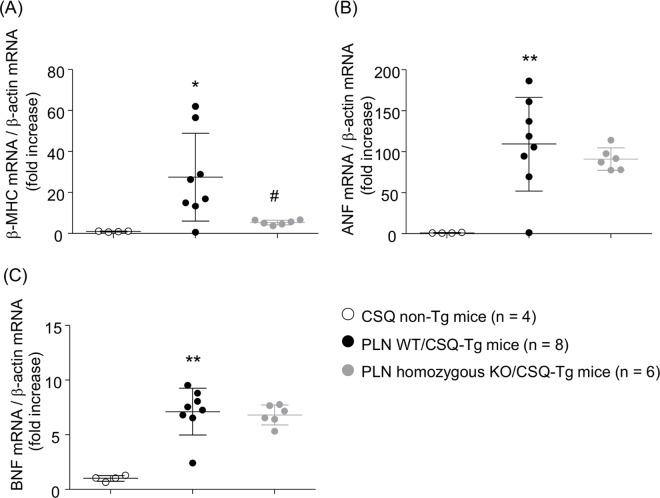
mRNA levels in the left ventricle. Analysis of mRNA levels was performed in the left ventricle of CSQ non-Tg mice, PLN wild type (WT)/CSQ-Tg mice, and PLN homozygous KO/CSQ-Tg mice. (A) β-MHC, (B) ANF, (C) BNF. Values represent the mean ± SD, *P < 0.05, **P < 0.01 vs. CSQ non-Tg mice, ^#^P < 0.05 vs. PLN WT/CSQ-Tg mice by Student's *t*-test.

To examine whether increase in ventricular weight by PLN ablation in CSQ-Tg mice is a pathological hypertrophy or not, we measured left ventricular mRNA levels of fetal genes, including β-MHC, ANF and, BNF, which were shown to be increased in pathological hypertrophy. All of three mRNAs were elevated in PLN WT/CSQ-Tg mice compared to CSQ non-Tg mice, associated with increase in ventricular weight (Fig 7). PLN ablation significantly inhibited the elevation of mRNA levels of β-MHC and did not show further increase of mRNA levels of ANF and BNF, in contrast to the results of left ventricular weight.

## Discussion

PLN inhibition and SERCA2a activation are known to show beneficial effects on cardiac function and hypertrophy in various HF models [17–19], and increase contractility in the cardiomyocytes isolated from heart failure patients [30], although there are few reports with respect to improvement of HF mortality [28, 29]. In this study, we examined whether PLN deletion can improve survival rate in CSQ-Tg mice. Consistent with previous report [22], CSQ-Tg mice showed premature death with cardiac dysfunction and hypertrophy and almost all of them died within 60 days after birth in this study. PLN deletion by CRISPR/Cas system significantly improved mortality, especially early death in CSQ-Tg mice. The probable cause of death in CSQ-Tg mice is considered to be pump failure, which results in insufficient blood supply to the organs of the body. In addition, pump failure produces thrombosis inside cardiac atria, which was often observed in CSQ-Tg mice and would be a cause of stroke death. PLN homozygous knockout showed a significant improvement of cardiac function in CSQ-Tg mice as previously reported [17], therefore it was thought that blood supply to whole body was increased and formulation of atrial thrombosis was inhibited, resulting in lowered mortality rate. Another possible cause of death is lethal arrhythmia. Arrhythmia is induced by abnormal Ca^2+^ homeostasis in HF [31, 32], and the increases in its incidence were observed in CSQ-Tg mice compared to CSQ non-Tg mice (data not shown). In the experiment of the measurement of cardiac function, we observed that PLN homozygous KO/CSQ-Tg mice showed less incidences of arrhythmia compared to PLN WT/CSQ-Tg mice (data not shown), suggesting that anti-arrhythmic effects of PLN ablation would also contribute to the improvement of mortality in CSQ-Tg mice. Conventional positive inotropic agents such as β-adrenoceptor agonists and PDE III inhibitors caused the worsening mortality in HF patients as a result of rising abnormal electrical activity and energetic consumption [20, 21]. However, PLN deletion and SERCA2a activation, which also has positive inotropic effect, did not increase HF death as shown in our study and had no harmful influences on energy consumption in the previous reports [33, 34]. PLN deletion did not affect blood pressure and heart rate, suggesting that afterload reduction and suppression of energy consumption were not involved in improvement of mortality in this study. Accordingly, PLN inhibition would be attractive approach to improve cardiac function with improved mortality unlike existing positive inotropic agents. While PLN deletion inhibited the premature death in this study, the death eventually caught up to that in PLN WT/CSQ-Tg mice. This result is inconsistent with another study that PLN deletion enhanced survival of β1-adrenergic receptor transgenic HF mice during one-year follow-up period [29]. One of the explanations of the discrepancy would be the limitation of CSQ-Tg mice as a model to examine agents or manipulations related to Ca^2+^ handling. PLN inhibition and SERCA2a activation increases Ca^2+^ uptake into SR, however, the increased Ca^2+^ inside SR could be caught up by highly expressed CSQ, a calcium binding protein, in CSQ-Tg mice, which might limit the efficacy of PLN inhibition and SERCA2a activation. In spite of this limitation, we used CSQ-Tg mice in this study because they are useful animal models for evaluating HF mortality with most of important features of HF [22, 23] described above. In addition, it has been reported that anti-HF drugs, such as angiotensin II receptor blocker, angiotensin converting enzyme inhibitor, and β blocker, can improve mortality in this animal model.

We confirmed complete deficits of PLN protein in PLN homozygous KO mice in this study. According to previous studies [35, 36], PLN ablation induced an increased SERCA activity and subsequent improvement of Ca^2+^ handling in mice, suggesting that our PLN homozygous KO showed similar effects on SERCA activity and intracellular Ca^2+^ handling. However, it remains to be determined whether these parameters are improved in our mouse model, which should be examined in future research.

Hypertrophy was developed in CSQ-Tg mice. PLN deletion significantly reduced atrial hypertrophy, but not ventricular hypertrophy. Atrial hypertrophy was inhibited by PLN deletion through the improvement of congestion, which is implied by the decreases in lung weight. In contrast, left ventricular weight was increased by PLN deletion. However, this increase in ventricular hypertrophy appears to be different from pathological hypertrophy, which is induced by pressure overload and volume overload in the HF, because mRNA of β-MHC, ANF and, BNF, which are increased in pathological hypertrophy, did not increase in PLN homozygous KO/CSQ-Tg mice. It is known that physiological hypertrophy induced by exercise training contributes to increase cardiac pump ability, and enables a greater supply of blood and oxygen to peripheral tissues, and differs in the structural and molecular profiles from pathological hypertrophy associated with pressure or volume overload [37, 38]. In CSQ-Tg mice, PLN ablation induced physiological hypertrophy followed by enhanced cardiac function, which counteracted pathological hypertrophy. Additional researches are required to verify this hypothesis.

In this study, we utilized the CRISPR/Cas system to generate PLN KO/CSQ-Tg mice. CSQ-Tg mice are known to have strain-specific variation [24, 25]. CSQ-Tg mice in this study have a complicated genetic background that crossed male DBA/2 overexpressed CSQ and female normal C57BL/6 mice for stable premature death. Due to this complicated background, it takes more than 2 years to obtain strain-matched knockout animals in conventional method of homologous recombination using ES cells because several times of crossbreeding are required. CRISPR/Cas system is a novel genome engineering technology, and is used for gene ablation in various species, such as mouse, rat, zebrafish, and pig by simplified design of guided RNA [39–43]. Although this technique was nearly applicable for normal animal so far, we applied it to disease model with a complicated genetic background and obtained PLN KO/CSQ-Tg mice in a very short period (about 3 months) from the IVF to the genotyping. Yield of transferred embryos and pups in CSQ-Tg mice were similar to normal mice, in addition, the knockout rate of PLN in CSQ-Tg mice with complicated genetic background is also comparable to that of PLN in normal C57BL/6 mice (data not shown). While an off-target effect is known as a limitation of this method [44, 45], we examined whether this CRISPR/CAS system produce the potential off-target effects in our study. As shown in S1 Fig, there are no deletions of six potential off-target sites identified by homology search (S1 Table) on PCR and sequencing analysis in PLN WT/CSQ-Tg mice and PLN homozygous KO/CSQ-Tg mice. This data suggests that CRISPR/CAS system showed a high specificity for PLN ablation in this study. However, we could not completely exclude the possibility of off-target effects induced by CRISPR/CAS system because there are still some possibilities of the mosaic off-target. These results suggest that CRISPR/Cas system is very useful to generate knockout mice even though it is difficult strain to establish the ES cells, or difficult disease model to naturally breed due to various reasons, such as short life span or obesity. Furthermore, even though mice do not have a complicated genetic background like CSQ-Tg mice, CRISPR/Cas system is beneficial, because homozygous deletion can be obtained by only one-time injection. This method could dramatically shorten the period to generate KO animals while we need to pay attention to high production rate of mosaic animal or off-target effect. However, the mice used in this study were not be backcrossed, thus they should be heterologous and have different gene expressions including potential different off-targets. This limitation should be considered when interpreting the results in our study.

In summary, PLN ablation significantly improved the mortality in the CSQ-Tg mice with increasing cardiac function and inhibiting hypertrophy, suggesting that PLN inhibition would be expected to be an attractive therapeutic approach to improve both cardiac function and mortality.

## Supporting Information

S1 Fig On-target and off-target analysis by PCR and sequencing. (A)(E) PCR products of 5' and 3' target site were not detected in PLN homozygous (Homo) KO/CSQ-Tg mice (n = 3), and were detected in parental PLN wild type (WT)/CSQ-Tg mice (n = 3). Sequencing data of the PCR products in WT/CSQ-Tg mice had a single signal. This means that parental WT mice do not have any mutations around the 5' and 3' sgRNA target sites. (B)-(D), (F)-(H) PCR and sequencing analysis of each potential off-target site in PLN Homo KO and PLN WT/CSQ-Tg mice. Three potential off-targets for 5' and 3' Pln sgRNA are shown in S1 Table. NGG PAM sequences are underlined and important 12 bases at each 3' end are capitalized. The capitalized sequences include up to 1 bp mismatch with each targeting sequence. Potential off-target sites were identified by homology search provided using NCBI BLAST at www.ncbi.nlm.nih.gov. Three off-target sites for each sgRNA target sequence were chosen in accordance with similarity to each sgRNA targeting sequence. In about 400 to 800 bp fragments from those off-target sites were amplified by PCR and directly sequenced. The following cycle conditions were used for PCR: 98°C for 10 s, 60°C for 15 s and 68°C for 10 s/kb for 32 cycles. The PCR products were analyzed by electrophoresis using LabChip GX system (Caliper LifeSciences, Massachusetts, USA). PCR products were treated with ExoSAP-IT (Affymetrix, California, USA) and verified by Sanger sequencing. In all six potential off-target sites, PCR fragments showed single bands and sequencing data showed single signals. These results indicate a high specificity of CRISPR/Cas system in this study. OFT is an abbreviation for potential off-target.
(TIF)

S1 Table Two sgRNA targeting sequences and those potential off-target sequences. OFT and chr are abbreviations for potential off-target and chromosome.
(DOCX)
