# Supplementary figures and images for: Phospholamban Ablation Using CRISPR/Cas9 System Improves Mortality in a Murine Heart Failure Model
Source: PLoS One. 2016 Dec 16;11(12):e0168486. doi: 10.1371/journal.pone.0168486 (PMC5161475; doi:10.1371/journal.pone.0168486)

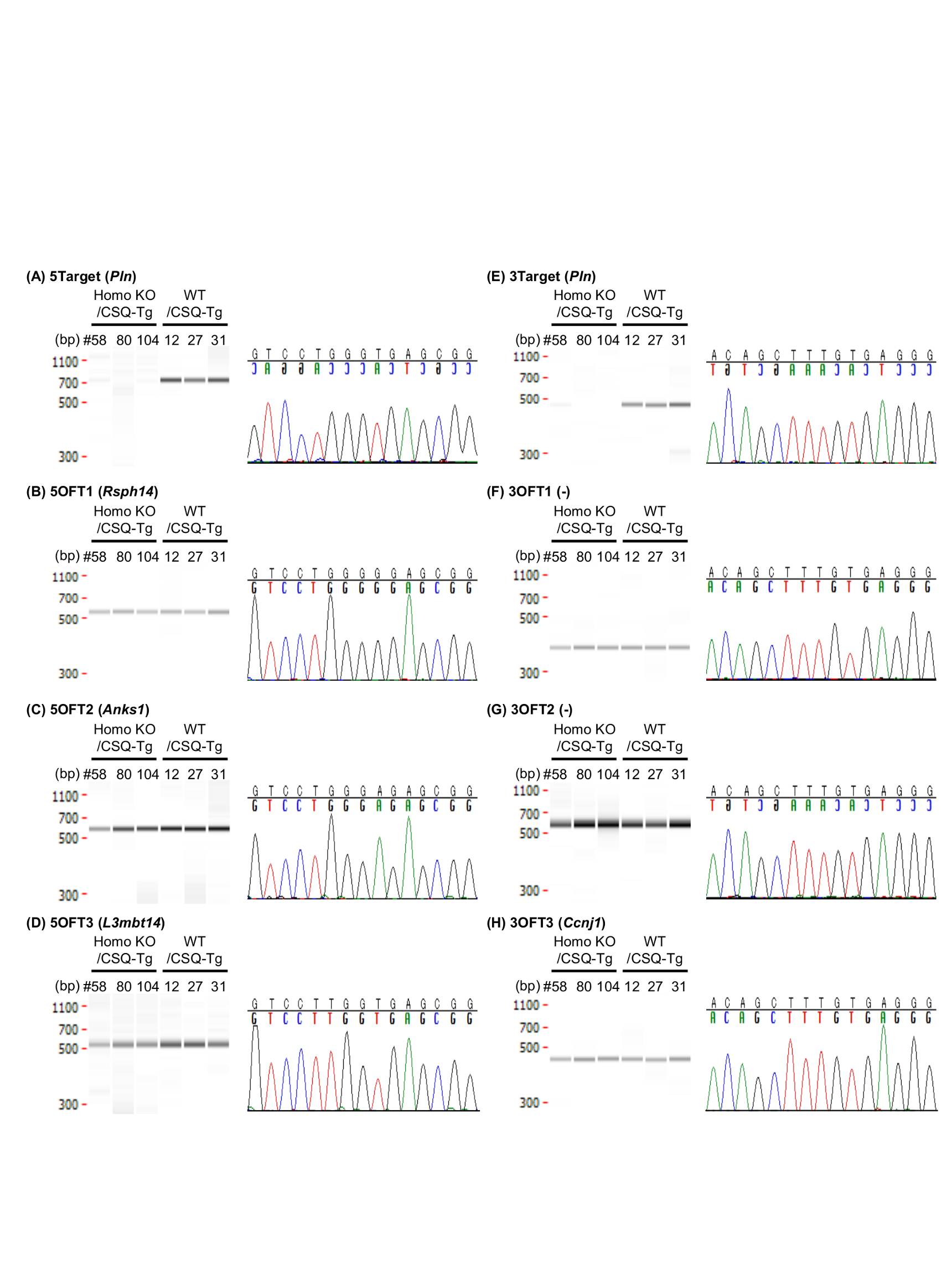

Supplement: S1 Fig — (A)(E) PCR products of 5' and 3' target site were not detected in PLN homozygous (Homo) KO/CSQ-Tg mice (n = 3), and were detected in parental PLN wild type (WT)/CSQ-Tg mice (n = 3). Sequencing data of the PCR products in WT/CSQ-Tg mice had a single signal. This means that parental WT mice do not have any mutations around the 5' and 3' sgRNA target sites. (B)-(D), (F)-(H) PCR and sequencing analysis of each potential off-target site in PLN Homo KO and PLN WT/CSQ-Tg mice. Three potential off-targets for 5' and 3' Pln sgRNA are shown in S1 Table. NGG PAM sequences are underlined and important 12 bases at each 3' end are capitalized. The capitalized sequences include up to 1 bp mismatch with each targeting sequence. Potential off-target sites were identified by homology search provided using NCBI BLAST at www.ncbi.nlm.nih.gov. Three off-target sites for each sgRNA target sequence were chosen in accordance with similarity to each sgRNA targeting sequence. In about 400 to 800 bp fragments from those off-target sites were amplified by PCR and directly sequenced. The following cycle conditions were used for PCR: 98°C for 10 s, 60°C for 15 s and 68°C for 10 s/kb for 32 cycles. The PCR products were analyzed by electrophoresis using LabChip GX system (Caliper LifeSciences, Massachusetts, USA). PCR products were treated with ExoSAP-IT (Affymetrix, California, USA) and verified by Sanger sequencing. In all six potential off-target sites, PCR fragments showed single bands and sequencing data showed single signals. These results indicate a high specificity of CRISPR/Cas system in this study. OFT is an abbreviation for potential off-target. (TIF) [file pone.0168486.s001.tif]
