# Supplementary material for: Phospholamban Ablation Using CRISPR/Cas9 System Improves Mortality in a Murine Heart Failure Model
Source: PLoS One. 2016 Dec 16;11(12):e0168486. doi: 10.1371/journal.pone.0168486 (PMC5161475; doi:10.1371/journal.pone.0168486)
Supplement: S1 Table — OFT and chr are abbreviations for potential off-target and chromosome. (DOCX) [file pone.0168486.s002.docx]

S1 Table. Two sgRNA targeting sequences and those potential off-target sequences.

| Name | Chr No. | Sequence | Gene Symbol |
| --- | --- | --- | --- |
| 5Target | chr10 | gtctcactGTCCTGGGTGAGcgg | *Pln* |
| 5OFT1 | chr10 | ccctccctGTCCTGGGGGAGcgg | *Rsph14* |
| 5OFT2 | chr17 | tggcagctGTCCTGGGAGAGcgg | *Anks1* |
| 5OFT3 | chr17 | aaccgcctGTCCTTGGTGAGcgg | *L3mbt14* |
| 3Target | chr10 | ggctgagaACAGCTTTGTGAggg | *Pln* |
| 3OFT1 | chr2 | ctagagaaACAGCTTTGTGAggg | *-* |
| 3OFT2 | chr11 | gtcagcacACAGCTTTGTGAggg | *-* |
| 3OFT3 | chr11 | aaagtcacACAGCTTTGTGAggg | *Ccnj1* |
